# Reimplantation of an autoamputated ovary in the omentum: A case report

**DOI:** 10.4274/tjod.24022

**Published:** 2015-12-15

**Authors:** Işın Üreyen, Derya Akdağ Cırık, Alper Karalok, Nazlı Türkcan, Taner Aksoy, Osman Türkmen, Nurettin Boran, Taner Turan, Gökhan Tulunay

**Affiliations:** 1 Antalya Training and Research Hospital, Clinic of Gynecology and Obstetrics, Gynecologic Oncology Division, Antalya, Turkey; 2 Etlik Zübeyde Hanım Women’s Health Training and Research Hospital, Clinic of Gynecologic Oncology, Ankara, Turkey

**Keywords:** Autoamputated ovary, reimplantation, revascularization

## Abstract

Autoamputation is one of the complications of ovarian torsion. In many cases, ovarian torsion develops as a result of mature cystic teratoma. Herein, we present a woman aged 27 years whose right ovary was autoamputated and reimplanted in the omentum. It should be noted that autoamputated ovaries can reimplant in surrounding tissues by revascularization and present as mobile abdominal masses with atypical localization.

## INTRODUCTION

Mature cystic teratoma is one of the frequently encountered ovarian tumors, and constitutes 5 to 25% of all ovarian tumors^(1)^.

Autoamputation is one of the complications of ovarian torsion^(2)^. In many cases, ovarian torsion develops as a result of mature cystic teratoma^(2)^. Reimplantation following autoamputation is possible with neovascularization and subsequent reperfusion, and thus the ovary can be resupplied.

In this article, we present a rare case of an autoamputated ovary that had reimplanted in the omentum.

## CASE REPORT

A woman aged 27 years presented to the gynecologic oncology clinic of our hospital with symptoms of nausea, vomiting, and inguinal pain, which had become increasingly worse throughout the previous week. The patient’s history revealed that she had had intermittent inguinal pain, which had been treated with analgesics. A gynecologic examination revealed a soft, mobile, mass with an irregular surface, which was about 5x5 cm in size and located in the left adnexial area. Ultrasonographic (USG) examination revealed cystic formations containing solid areas sized 27x22 mm, 29x22 mm, and 37x28 mm, with heterogeneous echoes sized 68x40 mm in the left ovary. The right ovary could not be observed in USG examination. Routine blood tests and tumor markers were normal.

**Figure 1:**
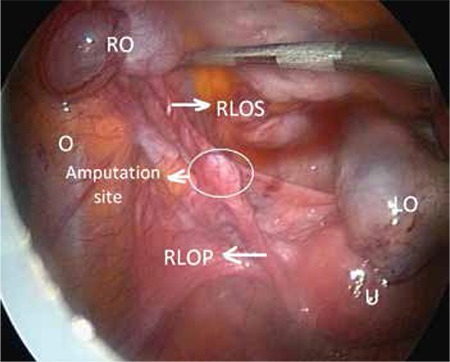
Uterus, left ovary and left fallopian tube. The right ovary was not observed at its original place O: Omentum, U: Uterus, LO: Left ovary, RO: Right ovary, RLOS: Right ligamentum ovary suspansorium, RLOP: Right ligamentum ovary proprium

**Figure 2:**
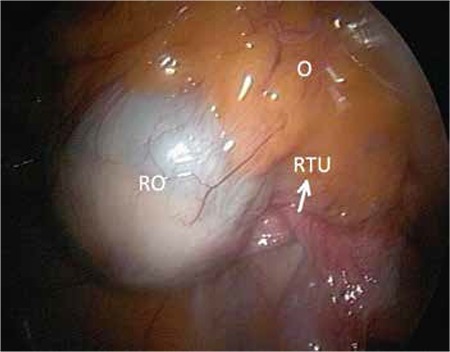
Reimplantation of autoamputated right ovary in the omentum O: Omentum, RO: Right ovary, RTU: Right tuba uterina

Laparoscopic observation revealed that the left ovary was multicystic; the right ovary and ampullary part of the right fallopian tube were not in place (Figure 1). The right ovary and distal part of the fallopian tube were found implanted in the omentum. The right ovary measured 5x4 cm and had a cystic structure (Figure 2).

**Figure 3:**
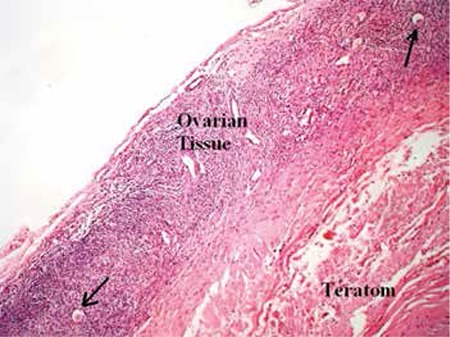
Ovarian tissue, primordial follicle is shown with arrow

**Figure 4:**
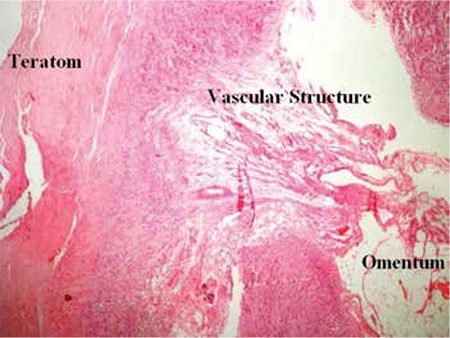
Ovarian tissue, omentum and vascular structure

The left ovary underwent cystectomy. The right ovary and distal part of the right fallopian tube, which were implanted in the omentum, were totally excised. Pathologic examination revealed mature cystic teratoma in both ovaries. The autoamputated ovary was found to have normal ovarian tissue (Figure 3). Revascularization was present between the right ovary and the omentum (Figure 4).

## DISCUSSION

Autoamputated ovary is an extremely rare gynecologic anomaly^(2,3)^. The prevalence is not fully known due to asymptomatic cases^(2,4)^. The diagnosis and treatment is not clear, because it is rarely seen. When these cases do arise, which is mostly during adolescence, the clinical presentation is acute abdominal pain due to ovarian torsion^(5)^. Further peritonitis and malignant transformation may rarely occur in patients who were asymptomatic during this period. Most patients can be diagnosed during an exploratory laparotomy^(2)^.

There are various theories to explain the formation of autoamputated ovary^(6)^. The first theory suggests that accessory or ectopic ovaries may occur due to the displacement of ovarian tissue after surgery or pelvic inflammatory disease. The second theory proposes that ectopic ovaries may occur due to abnormal sclerosis of migrating germ cells in the dorsal mesentery. A third possible theory is that ectopic ovaries form as a result of ovarian torsion due to ovarian cysts. The possible amputation mechanism of our case was torsion of the ovary, as mentioned in the third theory.

Vascular malnutrition and necrosis may develop due to ovarian torsion, resulting in autoamputation, which is a rare entity. In the majority of cases, autoamputation occurs in the right ovary because the sigmoid colon helps to prevent left ovarian torsion^(7)^. In our case, the right ovary was autoamputated, the left ovary had normal localization. The distal part of the right fallopian tube and ovary were implanted in the omentum.

The most frequent site of implantation of autoamputated ovaries was found to be the omentum^(2)^. The pelvis and cul de sac are other frequent areas of implantation. Intra-abdominal inflammation, adhesion formation, and subsequent reimplantation of the autoamputated ovary in the omentum occur after the ovarian torsion^(3,7)^.

Studies have shown the process of reimplantation and reperfusion following autoamputation^(8,9)^. In particular, the process of revascularization in grafts and vascular functionality were analyzed in patients with ovarian transplantation. It was observed that reperfusion was provided as a result of increased revascularization and angiogenesis in the recipient and graft tissues on the fifth day, and vascularization was significantly increased on the tenth day^(8,9)^. The potential target of angiogenesis of the recipient and graft tissues is to reduce the avascular period after transplantation^(8,9)^. Ovarian tissue was found in the amputated ovary in our case (Figure 3), which shows that the ovary was being supplied. In addition, the development of revascularization was observed between the ovary and omentum (Figure 4). The amputated ovary was being supplied by the revascularization in the omentum.

In conclusion, autoamputation of the ovary is a very rare entity. Its etiology is unclear and may be asymptomatic. Preoperative diagnosis is very difficult. It should be noted that autoamputated ovaries can reimplant in surrounding tissues by revascularization and present as mobile abdominal masses with atypical localization.
